# API2CAN: a dataset & service for canonical utterance generation for REST APIs

**DOI:** 10.1186/s13104-021-05593-w

**Published:** 2021-09-22

**Authors:** Mohammad-Ali Yaghoub-Zadeh-Fard, Boualem Benatallah

**Affiliations:** UNSW Sydney, Kensington, Australia

**Keywords:** Chatbots, Bot development, Natural language interfaces

## Abstract

**Objectives:**

Recently natural language interfaces (e.g., chatbots) have gained enormous attention. Such interfaces execute underlying application programming interfaces (APIs) based on the user's utterances to perform tasks (e.g., reporting weather). Supervised approaches for building such interfaces rely upon a large set of user utterances paired with APIs. Collecting such pairs is typically starts with obtaining initial utterances for a given API method. Generating *initial utterances* can be considered as a machine translation task in which an API method is translated into an utterance. However, the key challenge is the lack of training samples for training domain-independent translation models. In this paper, we propose a dataset for training supervised models to generate initial utterances for APIs.

**Data description:**

The dataset contains 14,370 pairs of API methods and utterances. It is built automatically by converting method descriptions of a large number of APIs to user utterances; and it is cleaned manually to ensure quality. The dataset is also accompanied with a set of microservices (e.g., translating API methods to utterances) which can facilitate the process of collecting training samples for building natural language interfaces.

## Objective

Recently natural language interfaces (also known as bots, chatbots, dialog systems, and virtual assistants) have attracted enormous attention because of their potentials in hands-free environments (e.g., self-driving cars) and accessibility products [1]. Building such interfaces often requires training utterances (e.g., *book a flight from Sydney to Paris*), annotated with user intents (e.g., book-flight) and associated parameters^1^ (e.g., from = “Sydney”, to = “Paris”) [1, 2]. Training utterances are used to train supervised models for detecting the users' intent based on their utterances. Because of the richness of human language, collecting large and diverse set of annotated utterances is required for building efficient natural language interfaces [2]. This is typically done in two steps: (i) generating an initial utterance (known as *canonical utterance*), and (ii) paraphrasing it to obtain more diverse training utterances [3–5]. In this paper, we focus on the first step, particularly for REST (Representational State Transfer) APIs since they are one of the most common forms of intents [6].

With the growing number of REST APIs and their ever-changing interfaces (e.g., renaming parameters, adding/removing operations), virtual assistants now require to automatically generate canonical utterances for scalability [4, 5]. Research has shown the feasibility of leveraging supervised machine translation techniques to generate canonical utterances for REST APIs [6, 7]. Ironically, such machine translation approaches also require training datasets to learn how to map REST API operations to canonical utterances. In this paper, we introduce the API2CAN dataset containing a large set of REST API operations (e.g., *GET /series/{id}/actors*) paired with their corresponding canonical templates (a canonical utterance in which parameter values have been replaced with placeholders e.g., *“get the list of actors of the TV series with id being* < *id* > *”).*

## Data description

To generate the *“API2CAN dataset”,* we obtained OpenAPI specifications indexed in OpenAPI Directory [8]. OpenAPI specification is a standard documentation format for documenting the interface of a REST API, including its operations and their parameters. OpenAPI Directory is a Wikipedia for REST APIs and maintains OpenAPI specifications for a large number of REST APIs. We obtained the latest version of each API index in OpenAPI Directory, and totally collected 983 APIs, containing 18,277 operations in total. Finally, we generated canonical utterances for each of the extracted operations as explained in [6]. In short, we converted the summary or description (e.g., “…gets the [actor](\#/definitions/Actor) by id. …”) of operations (e.g., GET /actors/{id}) in three steps: (i) extracting a sentence starting with a verb (e.g., “gets the actor by id.”), (ii) converting the extracted sentence to an imperative form to (e.g., “get the actor by id.”), and (iii) injecting the parameters (e.g., “get the actor by id being < id > .”) Finally, we manually cleaned the automatically generated utterances to ensure quality of the generated canonical utterances. As such we generated the API2CAN dataset which includes 14,370 pairs of operations and their corresponding canonical utterances, ignoring operations without generated canonical utterances. Next, we randomly divided the generated dataset into three parts as summarized in Table 1.

**Table 1:** Overview of data files/data sets

Label	Name of data file/data set	File types (file extension)	Data repository and identifier (DOI or accession number)
Dataset 1	API2Can-train.json contains 13,029 canonical utterances for 858 APIs	JavaScript Object Notation (.json)	https://doi.org/10.6084/m9.figshare.13331564 [9]
Dataset 2	API2Can-validation.json contains 433 canonical utterances for 50 APIs	JavaScript Object Notation (.json)	https://doi.org/10.6084/m9.figshare.13332029 [10]
Dataset 3	API2Can-test.json contains 908 canonical utterances for 50 APIs	JavaScript Object Notation (.json)	https://doi.org/10.6084/m9.figshare.13331507 [11]
Data file 1	API2Can-Schema shows a sample JSON object representing a training sample in the datasets	Figure (.png)	https://doi.org/10.6084/m9.figshare.13332347 [12]
Data file 2	API2Can Service contain the python scripts for generating the datasets	a zip file containing python codes (.py)	https://doi.org/10.6084/m9.figshare.13332125 [13]

**Fig. 1:**
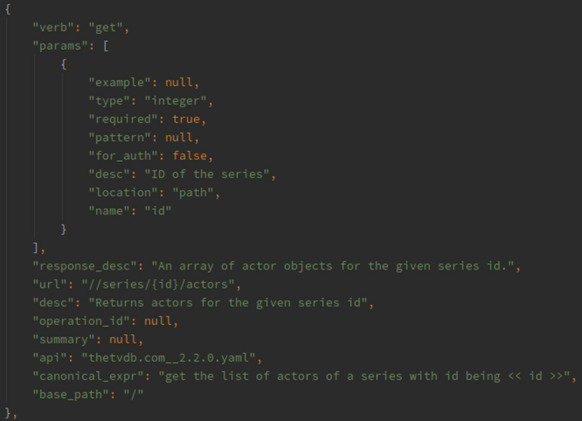
Dataset schema-sample operation [https://doi.org/10.6084/m9.figshare.13332347]

The API2CAN dataset is now public and accessible from [14]. The dataset is stored in a JSON (JavaScript Object Notation) array in which each element represents single Operation, including its API, and API version, endpoint (URL), HTTP verb (e.g., GET, POST), parameters, canonical utterances as shown in Fig. 1.

The generated canonical utterances can be used for training bots for existing REST APIs in the dataset. The API2CAN dataset can be also used to train machine translation systems to generate canonical utterances, which is required for new APIs.^2^ The API2CAN dataset is also accompanied with a set of microservices called *API2CAN Service*. The service is implemented as a standalone open-source REST Service in Python, and it is also accessible from [13]. This REST service provides several functionaries as follow:

- *Parsing OpenAPI Specification* This microservice parses the given API specification (in YAML format) and extract API elements such as operations and their parameters in JSON format.
- *Generating Canonical Utterances* This microservice generates the canonical utterances based on the approaches used in generation of the API2CAN dataset. In a nutshell, two approaches are used for generating canonical utterances as introduced in [6]: (i) converting operation summary as briefly introduced in the previous section, and (ii) *the resourced-based translator* which relays on the notion of Resources in REST APIs and is proposed in [6, 7].
- *Sampling Parameter Values* This microservice generates values (e.g., “Sydney”, “Paris”) for the parameters (e.g., “to”) of the given operation based on the approaches introduced in [6]. Generated values can be used to populate placeholders inside generated canonical utterances (e.g., “book a flight to Sydney”).

## Limitations

Given that fulfilling complex intents usually requires a combination of operations [4, 15], it is also needed to generate canonical utterances for compositions between operations. To achieve this, it is required to detect the relations between operations and generate canonical templates for complex tasks (e.g., tasks requiring conditional operations or compositions of multiple operations). Adding such cases thus require further research.

## Data Availability

The data described in this Data note can be freely and openly accessed on Figshare[9–14]. Please see Table 1 and reference list for details and links to the data.

## Abbreviations

API: Application Programming Interfaces
REST: Representational State Transfer
JSON: JavaScript Object Notation
URL: Uniform Resource Locator
HTTP: Hypertext Transfer Protocol

## Footnotes

1 also known as entities and slots.

2 APIs which are not included in the proposed dataset.
